# Minimally invasive buried guidance combined with stable orbital septal fat fixation for correction of tear trough-associated lower eyelid bags: application and outcomes

**DOI:** 10.3389/fmed.2025.1672397

**Published:** 2025-10-10

**Authors:** Qiang Zhang, Fangjian Min, Hongfu Xia, Chong Gao, Jie Song, Lei Jin, Wang Liu

**Affiliations:** ^1^The Department of Plastic Surgery, The Affiliated Hospital of Yangzhou University, Yangzhou University, Yangzhou, Jiangsu, China; ^2^Tianjin Key Laboratory of Retinal Functions and Diseases, Tianjin Branch of National Clinical Research Center for Ocular Disease, Eye Institute and School of Optometry, Tianjin Medical University Eye Hospital, Tianjin, China; ^3^Department of Plastic Surgery, University Hospital Münster, Münster, Germany

**Keywords:** lower eyelid bags, tear trough deformity, lower blepharoplasty, buried-guiding technique, orbital septal fat repositioning

## Abstract

**Background:**

Traditional transconjunctival lower blepharoplasty procedures that remove only the orbital septal fat often fail to effectively correct tear trough deformities, and may even exacerbate lower eyelid hollowness. Thus, developing a comprehensive surgical technique that can simultaneously correct both eye bags and tear troughs holds great clinical significance.

**Objective:**

This study aimed to evaluate a modified transconjunctival lower blepharoplasty technique that incorporates orbital fat repositioning and minimally invasive buried-guiding fixation to simultaneously correct eye bags and tear trough deformities. The goal was to restore a youthful contour of the lower eyelid and assess the clinical efficacy and safety of this approach.

**Methods:**

This study was designed as a prospective, single-center cohort study. A total of 30 patients (3 males, 27 females; mean age: 28 years) with lower eyelid bags and tear trough deformities were enrolled in this study. These patients were treated at the Department of Plastic Surgery, Affiliated Hospital of Yangzhou University, between January 2022 and December 2024. All patients underwent transconjunctival lower blepharoplasty combined with a buried-guiding fixation technique to perform strong orbital fat anchoring and correct both deformities.

**Results:**

All 30 procedures were successfully completed with primary healing achieved. The average follow-up period was 6.2 months (ranging from 3 to 12 months). Postoperative outcomes showed significant improvement in both lower eyelid bags and tear troughs, with a more youthful midface appearance and no external scarring. Patient satisfaction was high.

## Introduction

With aging, degenerative changes in the orbital fat and septal fascia of the lower eyelid commonly lead to sagging tissues and the appearance of protruding lower eyelid skin, often referred to as “eye bags” or “lower eyelid bags.” These changes result in a loss of smooth facial contours and a prematurely aged appearance (1). The formation of eye bags is frequently accompanied by tear trough deformities and, in some cases, midface depression (2). Prominent signs of aging in the lower periorbital region include orbital fat prolapse, tear trough depression, and deformities at the lid-cheek junction, collectively contributing to a fatigued, unhealthy, and prematurely aged appearance (3).

With the improvement of living standards, people have become increasingly concerned about their appearance, leading to a growing number of patients seeking lower eyelid bag correction surgery (3, 4). The demand is not exclusive to women, as a notable proportion of men also have concerns and seek improvement for lower eyelid bags (5). Lower blepharoplasty is one of the most common cosmetic surgical procedures but also among the more technically demanding (1). Based on the location of the surgical incision, lower eyelid procedures can be categorized into transcutaneous and transconjunctival approaches. For younger patients with little to no skin laxity, the transconjunctival approach is generally preferred, as they are less likely to accept the potential scarring associated with the transcutaneous method (2, 6, 7).

However, for patients presenting with lower eyelid bags combined with tear trough deformities, traditional transconjunctival blepharoplasty that solely removes orbital septal fat fails to achieve a smooth and youthful lower eyelid contour. Instead, it may exacerbate hollowing in the lower eyelid region (8, 9). Additionally, the limited surgical field makes it technically difficult to reposition and secure orbital fat for tear trough correction using the transconjunctival method (6, 10).

**Figure 1:**
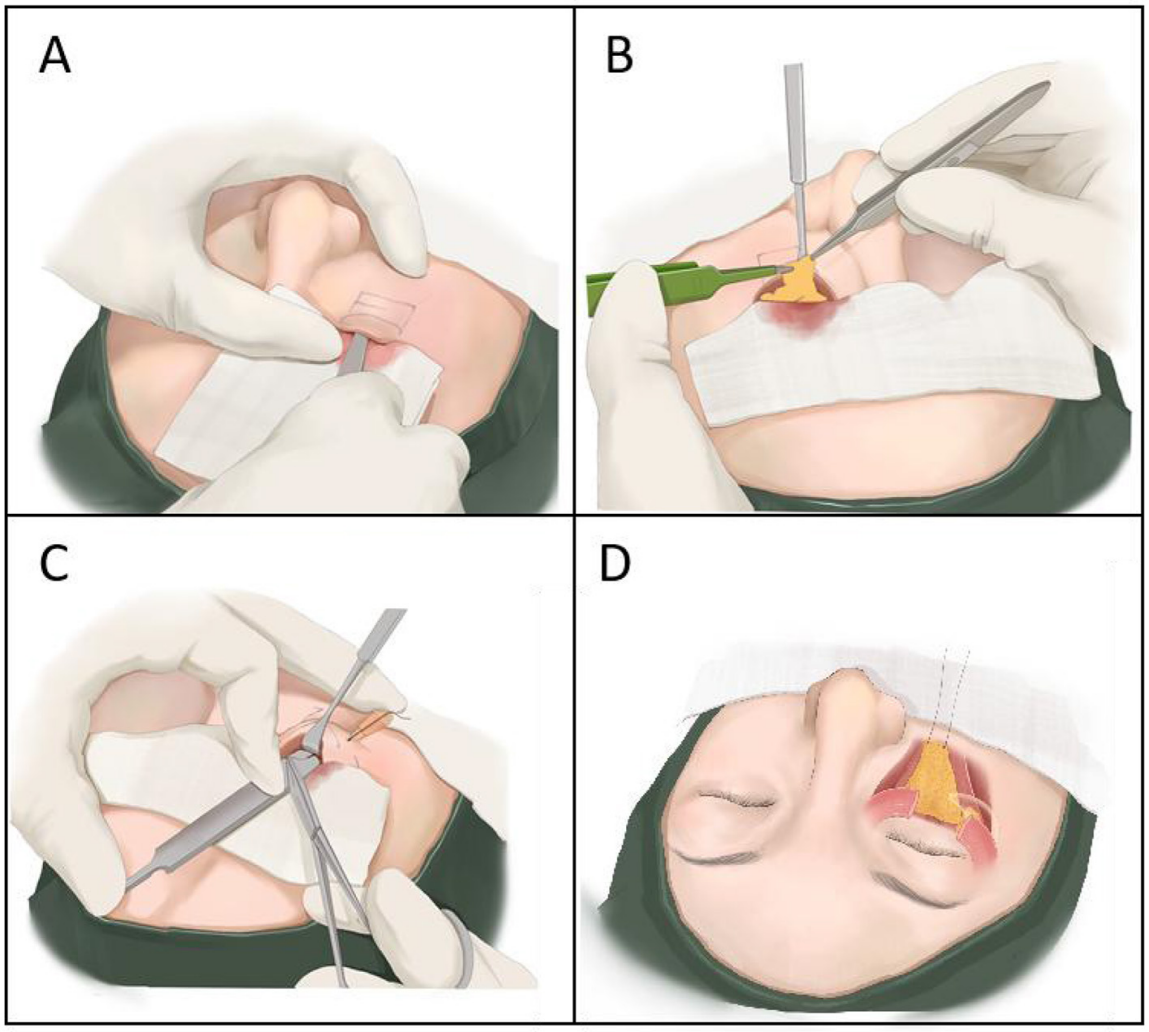
Main surgical steps for transconjunctival correction of tear trough deformity with fat repositioning: **(A)** Blunt dissection of premaxillary and prezygomatic spaces to release the tear trough and lid-cheek groove; **(B)** Creation of a tongue-shaped pedicled fat fascial flap; **(C)** Guiding needle insertion and external fixation of the fat flap using the suture tail; **(D)** External projection of the fixed fat flap.

Based on current clinical studies and experience (2, 3), we adopted a novel minimally invasive buried guidance technique via the transconjunctival approach to correct tear trough deformities. In this new method, we fully utilize the shape advantage of a wide handle dissector (Shanghai Jinzhong Instrument Co., Ltd., Model J11010, Shanghai, China) in combination with the anatomical characteristics of the infraorbital region to perform blunt dissection of the premaxillary and prezygomatic spaces under blind guidance. Then, with the assistance of lower eyelid retractors (Hangzhou Dino Medical Instrument Co., Ltd., 12 mm, Hangzhou, China), dissectors, and a 27-gauge guiding needle (Jiangsu Kangjin Medical Equipment Co., Ltd., Model 0.5 × 38RW LB, Jiangsu, China) (as shown in Figure 1), the prepared fat flap in the infraorbital region is guided and externally fixed.

## Data and methods

### General information

This study was designed as a prospective, single-center cohort study evaluating the clinical outcomes of a modified transconjunctival lower blepharoplasty technique that incorporates minimally invasive buried-guiding fixation for orbital fat repositioning.

This study included 30 patients (3 males and 27 females; age range: 25–32 years, mean: 28 years) diagnosed with lower eyelid bags combined with tear trough deformities. All patients had normal preoperative health check-ups, with no underlying medical conditions and no history of long-term medication use. The patients were treated between January 2022 and December 2024 at the Department of Plastic Surgery, Affiliated Hospital of Yangzhou University. All patients presented with protrusion of orbital fat and bilateral tear trough deformities, with either no or only mild skin laxity.

The study was approved by the Ethics Committee (Approval No. 2022-YKL1-20-013), and all patients provided informed consent to undergo transconjunctival lower blepharoplasty with correction of tear trough deformity. They also consented to pre- and postoperative photography and clinical follow-up.

### Surgical technique

#### Preoperative design

With the patient in a standing position, the degree of orbital fat protrusion and tear trough severity was assessed. A surgical marker was used to outline the area of fat bulge and the lower boundary of the tear trough. The design of the fat flap and its fixation site was determined based on clinical evaluation and patient preference (2, 11).

#### Surgical procedure

(a) The patient is placed in the supine position. Routine skin disinfection and draping are performed. Local anesthesia is administered using a mixture of 10 ml 2% lidocaine and 0.3 ml epinephrine for infraorbital and zygomatic nerve blocks, along with local infiltration anesthesia in the bilateral lower eyelid conjunctiva.

(b) Using a No. 11 blade (Shanghai Jinhwan Medical Instrument Co., Ltd., Shanghai, China), a 1.5 to 2 cm incision is made along the conjunctival surface of the lower eyelid. The incision is placed approximately 1 to 2 mm below the lower eyelid plate. Orbital fat is exposed through the incision. The assistant holds the upper conjunctiva edge with ophthalmic forceps, while the surgeon holds the lower edge with plastic surgery forceps, retracting both edges up and down, respectively. An electrocautery device is used to dissect inward along the exposed preseptal space, gradually exposing part of the orbital septum.

(c) A retractor is used to deepen the dissection, pulling tissue obliquely upward to expose the adhesion between the orbicularis oculi muscle and orbital septum. Electrocautery is applied along this adhesion, taking care to protect muscle tissue. The dissection space is fully opened medially and laterally to fully expose the orbital septal fat and orbital septum, ensuring the integrity of the septum. A mosquito clamp holding gauze is used for blunt horizontal dissection along the space to fully expose the arcus marginalis.

(d) The orbicularis retaining ligament is clamped with a mosquito forceps, and a wet gauze is placed on the forehead. After slight traction of the forceps toward the head, the gauze is naturally placed under it. Using plastic surgery forceps, the orbital septal fat is pulled outward to fully expose the arcus marginalis. Electrocautery is used to incise the orbicularis retaining ligament and tear trough ligament on the periosteal surface beneath the arcus marginalis muscle. The retractor is used to elevate the periosteum, exposing the levator labii superioris muscle. The blunt end of blade handle (12 cm, without blade), employed as a wide handle dissector, is then inserted above the surface of the levator labii superioris muscle. Using the handle in this manner, blunt dissection is performed caudally to separate the premaxillary and prezygomatic spaces down to the tear trough and lid-cheek junction. The dissection is considered complete when the handle tip can move freely in a pendulum-like motion within the space, ensuring full release (Figure 1A).

(e) A mosquito clamp holds the medial corner of the orbital septum, flattening the area beneath. An electrocautery blade makes a horizontal incision approximately 3 mm below the arcus marginalis at the lower orbital septum level, lifting the septal membrane upward. The septal membrane is clamped and placed on wet gauze toward the head. The assistant pulls the exposed orbital fat vertically upward. Electrocautery is used to dissect horizontally in front of the orbital ligament, separating the fat from the ligament. The lateral and central fat compartments are pulled outward to expose the medial fat compartment. Electrocautery dissects the septum separating the medial and central fat compartments, exposing the inferior oblique muscle. The muscle edge is released with electrocautery, fully freeing the inferior oblique muscle. The medial fat compartment is pulled with mosquito forceps, and the root of the medial fat is fully released using electrocautery. According to the volume of the orbital septal fat, some lateral fat is removed appropriately, and the remaining fat is sutured and fused to form a “tongue-shaped” pedicled fat-fascia flap (Figure 1B).

(f) The fat flap is sutured and knotted with 6-0 absorbable sutures (Hunan Housheng Medical Devices Co., Ltd., 6-0 monofilament Nylon, Hunan, China), leaving the suture tail intact. The guiding needle is inserted from the skin at the lower boundary of the tear trough into the premaxillary space. The retractor exposes the field, and the suture tail is threaded through the buried guiding needle hole. The needle is then passed back through the skin to the preoperatively designed lower boundary of the tear trough (the distance between the needle entry points equals the horizontal length of the fat flap), and an external surgical knot is tied (Figure 1C). Surgical tape (3M) is used to fix the externalized suture on both sides of the nasal ala.

(g) Count all surgical instruments and dressings. Apply petrolatum gauze over the eye, cover with sterile dressing, and fix with an eye patch. The patient is then returned to the ward.

(h) The external fixation sutures are removed one week after the procedure.

#### Clinical evaluation and statistical analysis

##### Evaluation criteria

In this study, patients were examined in the standing position, and the degree of orbital fat protrusion and tear trough severity were initially assessed clinically. Additionally, the depth of the tear trough was objectively evaluated using a modified Barton grading system, with scores ranging from 0 to 4 (9, 12). This system provided a standardized measure to assess the severity of the deformity and guide surgical planning. The criteria are summarized in Table 1. At the final follow-up visit, patients were also asked to rate their satisfaction with the surgical outcome on a 5-point scale: 5 = Excellent, 4 = Good, 3 = Fair, 2 = No improvement, 1 = Worsened (9).

**Table 1:** Modified Barton grading system for tear trough depth (9, 13).

**Score**	**Definition**
0	Absence of any demarcation or line separating the anatomic structures
1	Mild, subtle shadowing that is present only medially, with an overall smooth transition between anatomic structures
2	Mild shadowing or line that extends along the length of the eyelid
3	Mild shadowing with a visible prominence that extends along the length of the eyelid
4	Severe shadowing with overhanging tissue and obvious step deformity

### Statistical analysis

All data were analyzed using SPSS version 20.0. Categorical variables were assessed using the chi-square (χ^2^) test, while continuous variables were analyzed using the independent-sample *t*-test. A *p-value* of less than 0.05 was considered statistically significant.

## Results

**Figure 2:**
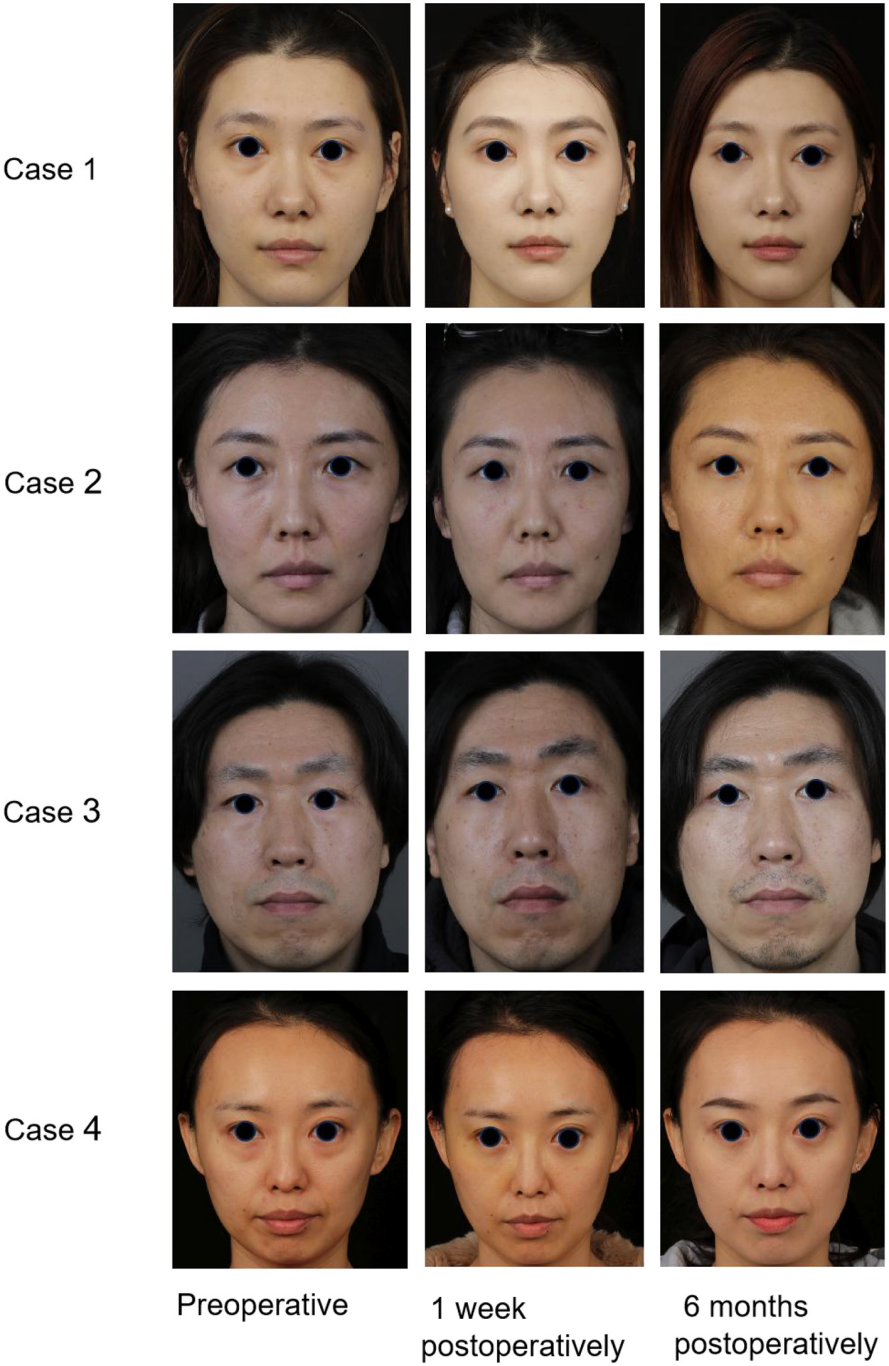
Case 1: A 30-year-old woman presented with a preoperative Barton score of 3, which improved to 0 postoperatively. Case 2: A 26-year-old woman improved from a Barton score of 2 to 0. Case 3: A 29-year-old man had a Barton score of 3 preoperatively and 0 postoperatively. Case 4: A 32-year-old woman had a Barton score of 4 preoperatively and 0 postoperatively. All four patients underwent transconjunctival orbital fat release, repositioning, and secure fixation for the correction of lower eyelid bags combined with tear trough deformities. Follow-up evaluations revealed marked improvement in both lower eyelid fullness and tear trough contour.

All 30 patients successfully underwent surgery with primary healing and no major complications such as diplopia, ectropion, or fat nodules in the grafted area. No patients were lost to follow-up during the observation period (Table 2). These patients were characterized by prominent lower eyelid bags and deep tear troughs. A transconjunctival approach with orbital fat release and repositioning was used to correct both deformities (Figure 2).

**Table 2:** Patient characteristics and procedure summary.

**Characteristics**	**No. (%)**
Age, Year (Average, Range)	28, 24–33
Sex (Female, Male)	3 (10%), 27 (90%)
Preoperative comorbidities	0
Follow-up duration, no. (Average, Range)	6.2, 3–12
**Postoperative complications**	
Eyelid ectropion/retraction	0
Diplopia	0
Fat nodules	0
Hematoma/edema	0
Infection	0

**Figure 3:**
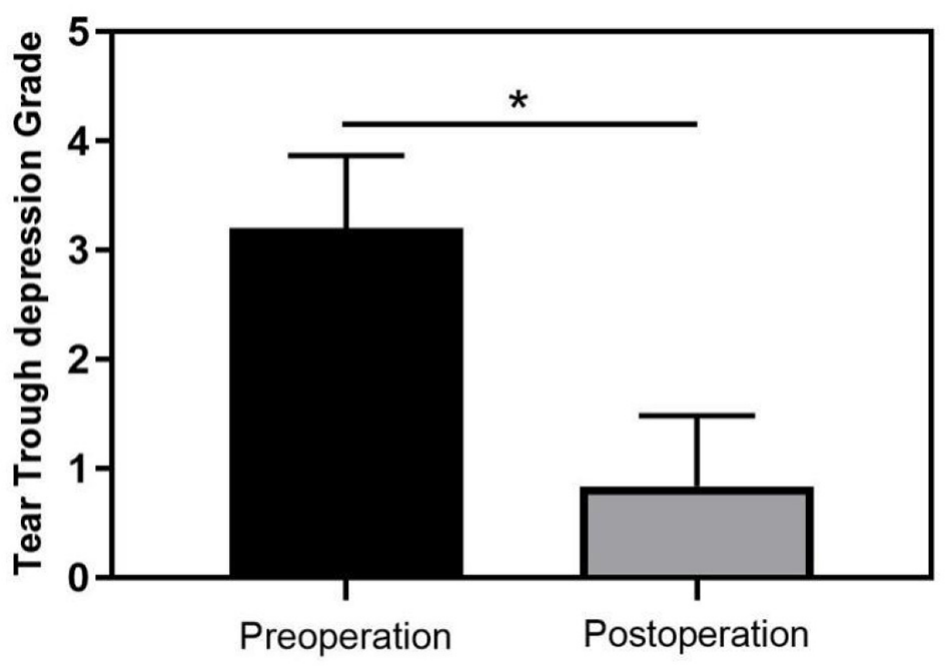
Change in tear trough depth before and after surgery (*p < 0.05 indicates statistical significance).

A comparative analysis of tear trough depression scores before and after surgery showed a significant improvement, with the mean preoperative grade decreasing from 3.2 ± 0.66 to 0.83 ± 0.65 postoperatively (*P* < 0.05; Figure 3). Among the participants, 18 (60.0%) rated their results as “excellent,” 10 (33.3%) as “good,” and only 2 (6.7%) as “fair.”

## Discussion

Changes in the eyelids and periorbital region are among the most prominent signs of facial aging and are often the primary concern for individuals seeking facial rejuvenation (4, 14). Lower blepharoplasty remains one of the most commonly performed procedures in orbital and periorbital rejuvenation surgeries (3, 4, 15). With aging, the orbital septum and overlying skin weaken, allowing orbital fat to herniate outward and form noticeable “eye bags” (16). Concurrently, due to the laxity of periorbital support structures, tear trough deformities often appear alongside eye bags (1). The tear trough represents a critical anatomic zone in the periorbital region and is a common manifestation of age-related orbital changes (17, 18). Its presence conveys a tired, aged, and unhealthy look, making its correction essential to aesthetic restoration (12, 14).

Over the past decade, the demand for rejuvenating the lower eyelid has increased dramatically, driven by a surge in procedures such as lower blepharoplasty and midface lifts (1, 19). Lower blepharoplasty remains a well-established surgical technique for addressing age-related changes in the lower eyelid, with a long track record of delivering satisfactory cosmetic results. In addition to correcting periorbital aging, the procedure often produces favorable improvements in midfacial contour (5, 8, 19).

Currently, lower eyelid blepharoplasty can be performed via a transcutaneous or transconjunctival approach. While both techniques are widely used, there is still no universal consensus on which method is optimal for specific deformity types or patient groups (19). However, there is growing agreement that preserving and repositioning orbital fat is a key surgical principle for both internal and external approaches to lower eyelid rejuvenation.

In this study, the patients were young individuals with lower eyelid bags and accompanying tear trough deformities, but without significant skin laxity. Given the minimal or mild skin laxity, we chose the transconjunctival approach, which avoids external incisions and scarring, reduces the risk of ectropion, and offers high patient satisfaction (9, 14). For older patients with significant aging and laxity, modifications may be required: adjunctive transcutaneous approach or skin excision for redundancy, more extensive release for weakened structures, and combination with skin pinch excision or resurfacing.

Traditional transconjunctival blepharoplasty typically involves simple excision of herniated orbital fat. While this may reduce protrusion, it often leads to increased hollowing in the tear trough and medial canthus regions, thereby exacerbating the deformity rather than correcting it (8, 9, 20). The current trend follows a philosophy of tissue preservation, which may include repositioning of the orbital and suborbicularis oculi fat (SOOF) as well as fat grafting to restore the noticeable volume loss associated with facial aging (2, 14, 15). Loeb emphasized that preserving and repositioning fat rather than excising it is effective in achieving a smooth and aesthetically pleasing transition between the lower eyelid and the cheek (21). Roh et al. (22) proposed that in patients with tear trough deformities, autologous fat grafting between the infraorbital skin and muscle layers can correct the deformity, and fat droplet injections at the same level can significantly improve (by 75%−90%) under-eye dark circles. Kim et al. (23) also concluded that tear trough deformities and translucent-type dark circles can be effectively treated with fat grafting. However, free fat grafts carry a higher risk of ischemia, which threatens the long-term survival of the transplanted fat and the durability of postoperative outcomes. Reports indicate that the absorption rate of free fat ranges from 20% to 90% (10, 24).

Therefore, it is now widely accepted that appropriately addressing tear trough deformities during lower eyelid surgery, when present, can lead to more favorable and long-lasting outcomes (1, 3). To address the limitations of free fat grafting in correcting tear trough deformities, Hamra further advocated for a modification of Loeb's technique by using a vascularized fat pedicle to fill the tear trough. Unlike conventional autologous fat grafting performed above the orbicularis oculi muscle, this approach utilizes well-perfused fat tissue to enhance long-term graft viability and durability (25). However, in transconjunctival fat repositioning procedures, the transconjunctival (internal) approach presents certain challenges due to limited surgical visibility and restricted operating space, which require a higher level of surgical expertise. Additionally, although dissection is performed beneath the orbicularis oculi muscle, securing and suturing the pedicled fat flap in place remains technically difficult (20).

Based on the original procedure, this study further refines techniques such as orbital septum release and fat transposition with stable fixation. By thoroughly releasing the tear trough ligament and its deep attachments, and separating the orbicularis oculi muscle from its periosteal insertion along the orbital rim, tissue forceps are used to access and dissect the premaxillary space. This anatomical plane lies between the deep layer of the orbicularis oculi muscle above and the superficial surface of the levator labii superioris muscle below. A pedicled fat flap is then securely anchored within this natural compartment without tension, achieving simultaneous correction of lower eyelid bags, improvement of tear trough deformities, midcheek augmentation, and overall midface rejuvenation (2, 26). By skillfully combining the use of dissectors, lower eyelid retractors, and a 27-gauge needle, we achieved a minimally invasive, buried guidance technique for the rapid and secure transposition and fixation of vascularized pedicled orbital septal fat flaps. This approach significantly reduces operative time. Moreover, compared to free fat grafting, the transfer of pedicled fat flaps ensures better vascular supply, providing a more favorable environment for graft survival (4, 26). Majidian et al. demonstrated that orbital septum fat repositioning effectively corrects tear trough deformities. Follow-up observations revealed no absorption of the pedicled fat grafts, indicating that the correction of the tear trough results from localized fat augmentation rather than fibrosis caused by fat necrosis (7). In our study, no fat induration was observed in the grafted area, and significant correction of the tear trough was achieved. Through optimization of intraoperative fat release and fixation techniques, a more rational distribution of orbital septal fat was attained, resulting in high patient satisfaction with postoperative appearance. During the procedure, the combination of dissectors and lower eyelid retractors was used to guide the needle, facilitating rapid localization and secure fixation of the fat flap. This approach holds promise for the future development of more advanced and practical instruments for orbital septum release and tear trough correction in lower eyelid surgery.

Subjective aesthetic outcomes are the cornerstone of cosmetic surgery (7, 9). In this study, we aimed to achieve high patient satisfaction using a modified transconjunctival lower blepharoplasty technique. Potential risks associated with the procedure include ectropion/retraction, diplopia due to inferior oblique muscle injury, fat nodules, hematoma/edema, and infection. To manage these risks, we employed strategies such as careful dissection, secure fixation, hemostasis, aseptic technique, and conservative management when necessary. Importantly, no major complications were observed in our cohort.

However, this study has several limitations. The absence of a control group prevents us from directly comparing our surgical outcomes with those of other techniques. As in previous studies, the evaluation of the effectiveness of periorbital surgery primarily relies on subjective feedback from both surgeons and patients regarding satisfaction. Moreover, the relatively short follow-up period limits our ability to assess the long-term efficacy and complications of the new technique. Additionally, the lack of imaging data, such as ultrasound, to objectively assess the long-term stability of the repositioned fat represents another limitation. Future studies will incorporate imaging techniques to provide more objective evidence of the long-term outcomes and stability of this technique.

## Conclusion

Our study demonstrates that transconjunctival lower eyelid blepharoplasty combined with orbital septal fat repositioning, involving separation of facial soft tissue planes and fat transposition to the tear trough deformity area, effectively corrects tear trough deformities and lower eyelid bags, achieving rejuvenation of the lower eyelid. The minimally invasive buried guidance technique simplifies the surgical procedure, allowing secure fixation of the transposed fat, which ensures graft survival and prevents fat resorption. Additionally, this method reduces complication risks and operative time, resulting in high satisfaction rates among both patients and surgeons, making it an ideal option for treating tear trough deformities.

## Data Availability

The original contributions presented in the study are included in the article/Supplementary material, further inquiries can be directed to the corresponding authors.
